# Gamma Correction and Color Space Transformations for Quantitative Analysis of Electrochemiluminescence Images Using Smartphone Cameras

**DOI:** 10.1021/cbmi.5c00056

**Published:** 2025-06-26

**Authors:** Stephania Rodríguez Muiña, Rajendra Kumar Reddy Gajjala, Eduardo Fernández Martín, Francisco Javier del Campo

**Affiliations:** † Basque Center for Materials, 518636 BCMaterials Applications and Nanostructures. UPV/EHU Parque Científico, Leioa, Bizkaia E-48940, Spain; ‡ Basque Foundation for Science, 197447 IKERBASQUE, Bilbao 48009, Spain

**Keywords:** smartphone, gamma correction, image analysis, electrochemiluminescence (ECL), optical biosensing, Colorimetry, signal quantification, color space transformations

## Abstract

Quantitative imaging of luminescent signals, ranging from electrochemiluminescence (ECL) and chemiluminescence to colorimetric assays, is increasingly performed using consumer-grade digital cameras and smartphones. However, device-dependent variability, nonlinear signal encoding, and the absence of standardized workflows hinder reproducibility and quantification accuracy. This work presents a generalized methodology for robust signal quantification in luminescent systems using digital imaging, with ECL as a model case. By combining synchronized electrochemical control, manual optimization of imaging parameters, gamma correction, and color space transformations, accurate device-independent analysis is enabled. Using Ru­(bpy)_3_^2+^/TPrA as a test system, we evaluate RGB, CIEXYZ, and CIELAB color spaces, identifying optimal channels for sensitivity and dynamic range. Our performance assessment underscores the importance of transfer function selection and supports both linear and nonlinear quantification models. Results show that linearized r and X color channels offer broad dynamic ranges with moderate sensitivity, while encoded R and a* channels provide higher sensitivity at low concentrations, requiring nonlinear modeling to extend their quantification range. This scalable approach enables standardized, high-throughput optical analysis using low-cost camera platforms, with broad applications in diagnostics, biosensing, and analytical chemistry.

## Graphical abstract

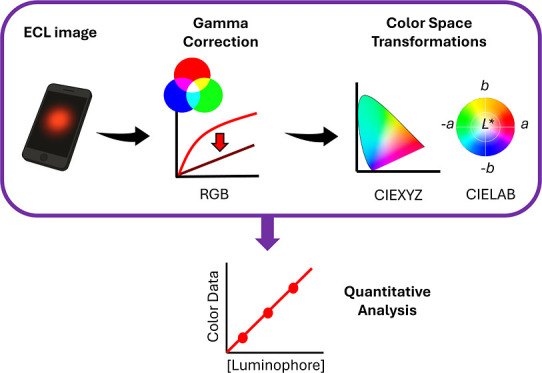

## 1 Introduction

Since the introduction of smartphone cameras for paper-based assay quantification by Whitesides et al. in 2008,^1^ their use has become widespread in scientific literature due to their portability, affordability, and high-resolution imaging capabilities.^2–14^ This has made smartphones an attractive alternative to laboratory-grade detectors in colorimetric,^5,8,12,13,15,16^ fluorescence,^5^ and chemiluminescence-based assays,^2,9,17^ particularly for point-of-care applications.

Electrochemiluminescence (ECL), a light emission phenomenon triggered in solution by electrochemical processes,^17–21^ is particularly suited to camera-based imaging because of its high sensitivity, spatial resolution, and negligible background.^2–6,11,12,15,17,22–30^ An important advantage of ECL and other luminescent systems is that the captured signal corresponds to the emitter spectral power distribution, eliminating the need to account for external illuminants, limiting the effort to keep consistent image focus and capture conditions. This is in contrast to reflective systems, where surface albedo and light source properties critically affect the signal, illumination conditions and viewing angle strongly influence observed color.^6,12,31–35^

ECL imaging emerged in the 1980s^26,27,30,36^ as a powerful alternative to spectrometers and photodetectors, enabling spatially resolved visualization of reaction kinetics, electrode heterogeneity, and mass transport phenomena.^15,23,26–30,36–38^ The evolution from monochrome sensors^15,26–30,36,38^ to CCD-based microscopes,^18^ and more recently to CMOS smartphone cameras,^5,6^ have significantly enhanced accessibility and broadened ECL experimental scope.^2–6,8,11,12,15,16,22–25^ However, proper handling of image data remains a major limitation.

In spite of the growing use of smartphone cameras, the lack of understanding of the digital image processing pipeline often leads to an incorrect interpretation of digital color data.^5,6,10,12,13,39–42^ A common misconception is the assumption that RGB values scale linearly with the concentration^14,43^ when, in reality, the tonal response curves in standard image formats are strongly nonlinear due to adjustments such as gamma correction.^5,10,33,40,42^

Figure 1a outlines the main image-processing steps that affect quantification accuracy. Briefly, after light passes through the camera optics, it encounters a Bayer filter array over the sensor. This color filter array separates the incident light into red (R), green (G), and blue (B) components by allowing each photosite to capture only one spectral band, typically with twice as many green filters as red or blue.^33,35^ The sensor converts the filtered light into a digital value, resulting in linear raw data.^33,35^ Its spectral sensitivity, represented in [Figure 1b­(1)], depends on both the filters and the detector itself^33^ and will differ between camera models.^44^

**Figure 1:**
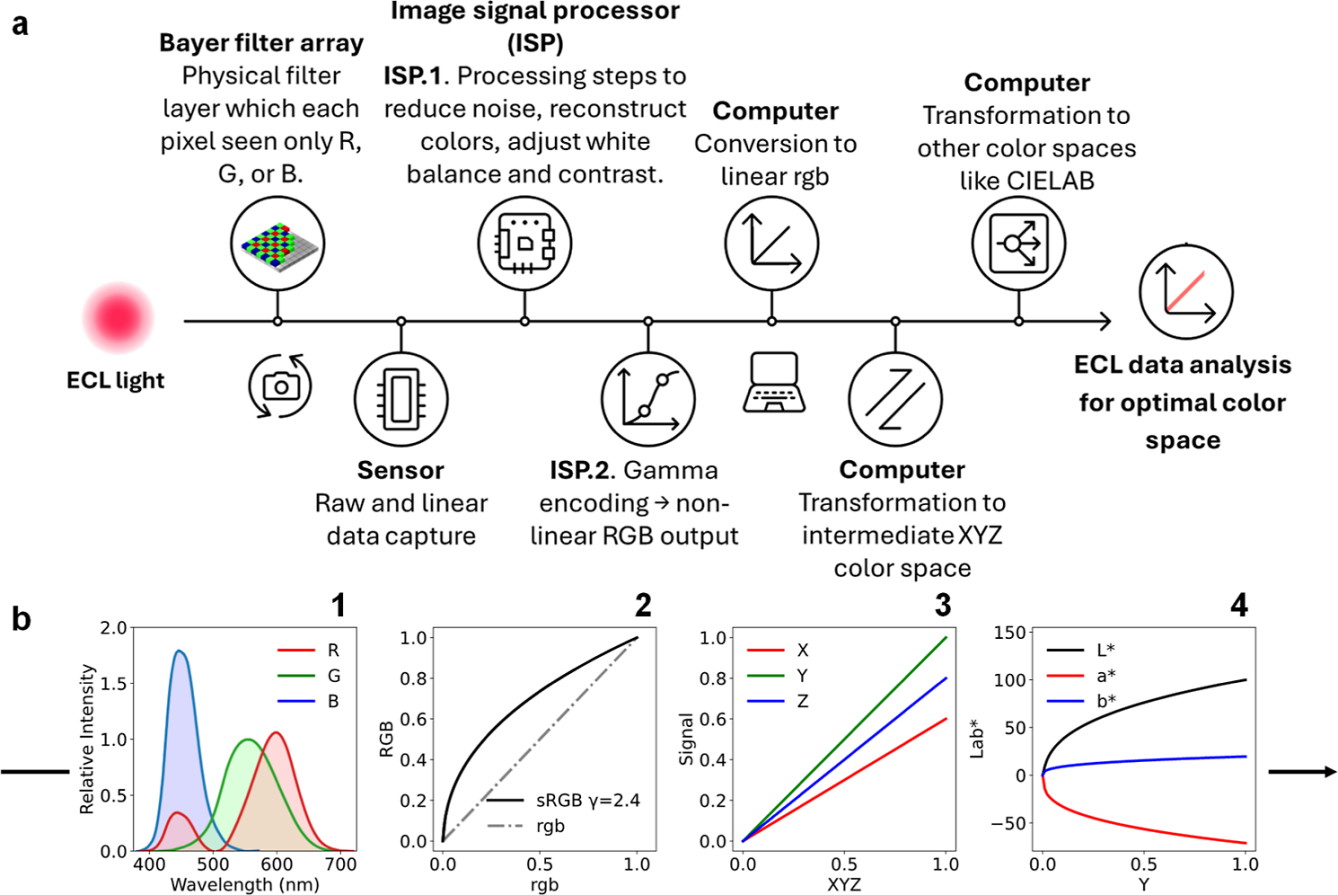
(a) ECL signal processing workflow illustrating the digital camera imaging pipeline and color space transformation steps for ECL quantifiable analysis. (b) Example of a data treatment pathway from the workflow, beginning with the spectral sensitivity of a typical color camera sensor (1), followed by sRGB color encoding (2), highlighting both the gamma-encoded curve (black line) and the gamma-corrected linear segment (gray dashed line). The data is then transformed to the intermediate CIEXYZ color space (3) and finally converted to the CIELAB color space (4) for perceptual uniformity visualization.

These data are then transformed by the image signal processor (ISP) through demosaicing, white balance, noise reduction, and signal amplification, each of which can alter the original signal.^33,35^ Gamma correction, a key nonlinear encoding step used to compress image data for formats like JPEG or MPEG,^33^ further complicates analysis by discarding perceptually redundant information.

Gamma encoding typically follows a piecewise function, with a linear segment near black level and a nonlinear exponential region.^33,45^ For quantitative analysis, these gamma-encoded values must be linearized (as represented in [Figure 1b­(2)]) to preserve the correct relationship between signal intensity and analyte concentration.^9,33,46^ Several studies have emphasized the risks of neglecting this step and the need to control image acquisition settings to ensure reliable and reproducible camera-based detection.^5,6,12,13,33–35,39,47,48^

To improve reproducibility and cross-platform consistency, image data can be transformed from gamma-encoded RGB into device-independent color spaces such as the linear CIEXYZ space and subsequently into perceptually uniform spaces like CIELAB,^5,33,45,46,48^ both represented in [Figure 1b­(3–4)]. Each of these spaces plays a distinct role in ensuring accurate signal interpretation. However, such transformations depend on knowledge of the original RGB encoding and gamma functions, which are often unspecified in compressed formats.

This work highlights the critical importance of controlling image acquisition parameters, accounting for gamma correction, and applying appropriate color space transformations in camera-based detection. Using the Ru­(bpy)_3_^2+^/TPrA ECL model system, we demonstrate a practical image-processing workflow (illustrated in Figure 1b) and compare the performance of RGB (sRGB and BT709), CIEXYZ, and CIELAB color spaces in terms of sensitivity, linear range, limit of detection (LOD), and limit of quantification (LOQ). Although demonstrated with ECL, the methodology could be adapted to colorimetric assays, where additional challenges arise from light reflection. This framework aims to improve the accuracy, reproducibility, and comparability of image-based analyses in chemical and biomedical research.

## 2 Materials and Methods

### 2.1 Reagents

Tris­(2,2′-bipyridine)­ruthenium­(II) hexafluorophosphate (Ru­(bpy)_3_^2+^, 97%, CAS 60804742), tripropylamine (TPrA, 98%, CAS 102692), sodium phosphate monobasic dihydrate (99%, CAS 13472350), sodium phosphate dibasic (99%, CAS 7558794) and phosphoric acid (85%, CAS 7664382) were used as obtained from Merck-Aldrich.

### 2.2 Instrumentation

Electrochemical measurements were carried out using a PalmSens4 potentiostat controlled by PSTrace 5.9 software on a Windows 10 PC. A conventional three-electrode setup was used, consisting of a set of five 3 mm glassy carbon disc working electrodes (BASi, West Lafayette, USA) that were alternately employed as the working electrode, along with a Hach 5057 combined electrode featuring an Ag/AgCl single-junction reference in 3 M KCl and a platinum (Pt) auxiliary electrode.

Glassy carbon working electrodes were polished to a mirror finish using alumina slurries with particle sizes of 0.3 and 0.05 μm. After polishing, electrodes were sonicated for 15 s in a water–ethanol mixture to remove residual particulates, rinsed thoroughly with deionized water, and dried under a compressed air stream.

Video recording of electrochemical experiments was performed using an Honor Magic 4 Lite 5G smartphone camera (48 MP, f/1.8, 27 mm) equipped with a macro lens (24× Apexel, Amazon, ES) synchronized with a PalmSens4 system through a customized Bluetooth wireless trigger (Comioke, Amazon, ES).^9^

### 2.3 Electrochemiluminescence Experiments

ECL measurements were performed in solutions containing 180 mM TPrA in 0.3 M phosphate buffer (pH 6.7)^20,49^ with Ru­(bpy)_3_^2+^ concentrations ranging from 0 to 250 μM. Multipotential step amperometry was conducted by applying 0 V (vs Ag/AgCl) for 5 s, followed by a step to 1.2 V for 10 s. Additionally, cyclic voltammetry (CV) was performed at selected concentrations by scanning from 0 to 1.5 V (vs Ag/AgCl) at a scan rate of 0.05 V s^–1^.

ECL emission was detected using a custom-built optoelectrochemical setup, schematized in Figure 2. The system featured a 3D-printed holder to ensure a precise camera-electrode alignment. The entire assembly was enclosed in a black box to avoid ambient light.

**Figure 2:**
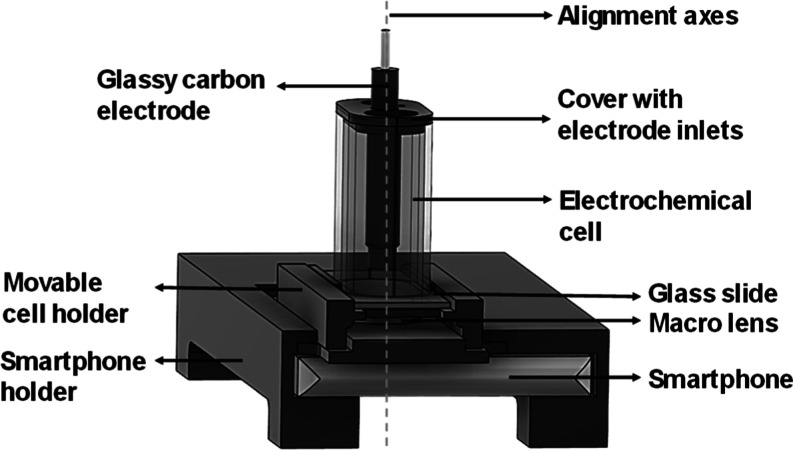
Schematic representation of the cell designed for ECL experiments. The setup includes a 3D-printed holder that aligns camera and working electrode. The electrochemical cell is mounted on a glass slide to allow imaging of the electrode surface. It includes dedicated inlets for electrode placement and a precision holder to maintain a fixed focal distance. A gap between the cell and the camera accommodates a macro lens for optimized image capture.

### 2.4 Raw Video Capture and Video Encoding: Smartphone End

Raw videos were captured using the *MotionCam Pro* application (version 3.5.5-pro, Play Store) installed on the Honor Magic 4 Lite running Android OS 11. This application allowed direct access to unprocessed sensor data.

Imaging parameters were optimized by a calibration process using the lowest and highest analyte concentrations to ensure an optimal dynamic range across conditions, preventing underexposure or overexposure. The following parameters were selected for recording: a resolution of 1920 × 1080 pixels, fixed focus, a frame rate of 1/5 s, and equivalent ISO 100 (further details can be found in Supporting Information Section S1.1).

Raw video data (MCRAW lossless format) was encoded using the H.265 (Hardware) codec with a BT709 transfer function and 8 bit depth (0–255 range). The full 2^8^-range capability was verified by recording highly reflective and dark surfaces. All rendering parameters, including saturation, contrast, and color temperature, were tightly controlled to ensure reproducibility across experimental conditions (Table S1).

### 2.5 Video Data Processing: Computer End

Video data were analyzed using custom Python scripts (available at GitHub branch for Gamma Correction and Color Space Transformations) that incorporated OpenCV along with additional scientific computing libraries such as NumPy and pandas. Individual video frames were extracted for automated processing, and a circular mask was applied to isolate the electrode region. Color intensity values were obtained by calculating the mean and standard deviation of the red, green, and blue channels within the masked electrode region for each BT709-encoded frame. Each video was then automatically paired with its corresponding electrochemical data series. This workflow enabled the simultaneous extraction of electrochemical current and ECL emission signals from both video and potentiostat data, streamlining the analysis of large data sets.

The brightest frame in each video, corresponding to peak ECL emission, was identified and used for further analysis. Data processing involved a sequence of steps including normalization, linearization using inverse optoelectronic transfer functions (OETFs), and encoding into alternative color spaces. Further details on these transformations are provided below.

Step 1: Normalization

First, data was normalized by scaling values to a 0–1 range through division by 255, corresponding to the tonal depth of 8 bit RGB.^5,10^

Step 2: Linearization—RGB Gamma correction

Second, BT709 RGB data was linearized by applying gamma correction. This function corresponds to the RGB inverse gamma transfer function (eq 1), which involves a piecewise function consisting of a linear region for tonal values below a certain threshold, followed by an exponential region.^33,45,46^ RGB data linearization shows the direct proportionality between the concentration of reacting species and light intensity.^9,33,46^

1 $$rgb=\{\begin{array}{l} \frac{RGB}{a},RGB<c\\ {\left(\frac{RGB+b}{1+b}\right)}^{\gamma },RGB\geq c \end{array}$$

where RGB is the normalized color channel encoded data and *rgb* is the linear tristimulus value.

For BT709 encoding factors *a*, *b* and *c* are 4.5, 0.099 and 0.081, while γ, the gamma value, is commonly 2.2.^46^ Nevertheless, gamma should be experimentally determined in each case, as it varies depending on the digital processing of the specific device. The experimental determination of gamma using LEGO bricks of known sRGB values has recently been described,^50^ but the procedure is summarized for convenience in the Supporting Information Section S1.3.

Step 3: Transformation to other color spaces: BT709 to CIEXYZ, sRGB and CIELAB

### 2.6 The Processed BT709 Color Data was Transformed to Analyze Other Color Spaces, including CIEXYZ, sRGB, and CIELAB

Transformation between color spaces began with converting linear *rgb* values to the intermediate CIEXYZ color space.^5,10^ CIEXYZ is a device-independent model that separates luminance (*Y*) from chromaticity components (*X* and *Z*), ensuring consistent and interoperable color representation across platforms, which facilitates accurate color analysis.^51^

The transformation was performed using a standard matrix relative to the D65 illuminant (eq 2), which served as the reference white in this study. For other illuminants, the matrix coefficients differ.^5,10,33,45,46,48^

2 $$\left[\begin{array}{l} X\\ Y\\ Z \end{array}\right]=\left[\begin{array}{lll} 0.4124 & 0.3576 & 0.1805\\ 0.2126 & 0.7152 & 0.0722\\ 0.0193 & 0.1192 & 0.9505 \end{array}\right]\left[\begin{array}{l} r\\ g\\ b \end{array}\right]$$

Conversion from CIEXYZ to sRGB can be mathematically performed using the inverse of the above transformation matrix.^33,46^ The full mathematical formulation is provided for completeness; however, this step was not necessary in the current workflow, as the original data were already encoded in the BT709 RGB color space. Instead, transformation was carried out directly from the linearized rgb values by applying the gamma-correction transfer function (eq 3, the inverse of the eq 1), followed by scaling the normalized sRGB values to the 0–255 range.^10,33,46^

3 $$RGB=\{\begin{array}{l} a\cdot rgb,\;rgb<c/a\\ {\left(1+b\right)rgb}^{1/\gamma }-b,\;rgb\geq c/a \end{array}$$

For sRGB encoding factors *a*, *b* and *c* are 12.92, 0.055, and 0.04045 for sRGB, respectively, and the applied gamma (γ) value was 2.4, commonly used for this space.^5,10,46^

Finally, the CIEXYZ values were transformed into the CIELAB color space for perceptual analysis. CIELAB, also known as *L* ^*^ *ab*, is designed to be perceptually uniform, meaning that numerical differences in this space correspond to perceptually equal color differences. Due to its ability to reflect human visual sensitivity to subtle color variations, CIELAB is widely used in colorimetric applications, such as quality control and digital imaging.^33,52^

This was done using eq 4,^33,52^ corresponding to the standard CIE 2004

4 $$L^{*}=116f\left(\frac{Y}{Y_{n}}\right)-16$$

$$a^{*}=500\left(f\left(\frac{X}{X_{n}}\right)-f\left(\frac{Y}{Y_{n}}\right)\right)$$

$$b^{*}=200\left(f\left(\frac{Y}{Y_{n}}\right)-f\left(\frac{Z}{Z_{n}}\right)\right)$$

$$with\;f\left(i\right),representing\left\{f\left(\frac{X}{X_{n}}\right),f\left(\frac{Y}{Y_{n}}\right)\text{or}\;f\left(\frac{Z}{Z_{n}}\right)\right\},calculated\;\text{as}:$$

f

$$\left(i\right)=\{\begin{array}{l} \frac{1}{3}{\left(\frac{29}{6}\right)}^{2}i+\frac{16}{116},i\leq {\left(\frac{6}{29}\right)}^{3}\\ i^{1/3},i>{\left(\frac{6}{29}\right)}^{3} \end{array}$$

Here, *X*_*n*_, *Y*_*n*_ and *Z*_*n*_ represent the tristimulus values of the CIE Standard Illuminant, for D65 they are 0.95047, 1, and 1.08883, respectively. The L* parameter represents lightness (ranges from 0 to 100), while a* and b* correspond to the red-green and yellow-blue chromatic axes, respectively, spanning from −100 to 100 ^33^ ^,^ ^52^.

These transformations facilitated more accurate color analysis, allowing for the evaluation of color variations in a device-independent manner.

## 3 Results and Discussion

### 3.1 Image-Based ECL Signal Dynamics

Electrochemical techniques, including cyclic voltammetry and multistep amperometry, were applied as described in Section 2.3, while videos of the electrode surface were recorded in real-time to monitor the electrode response.

The ECL mechanism of Ru­(bpy)_3_^2+^/TPrA system has been extensively studied and reported in the literature.^21,53–56^ Briefly, it involves both electrode and solution-phase reactions. Ru­(bpy)_3_^2+^ and TPrA are oxidized at the electrode, with TPrA forming a radical cation (TPrA^•+^) that deprotonates to yield the strongly reducing TPrA^•^ radical. This radical can reduce either Ru­(bpy)_3_^3+^ or Ru­(bpy)_3_^2+^ to generate the excited state Ru­(bpy)_3_^2+^, which emits light at ∼620 nm. In addition to the classical oxidative–reduction pathway, a solution-phase route involving Ru­(bpy)_3_^+^ and TPrA^•+^ can also lead to Ru­(bpy)_3_^2+^ formation, accounting for ECL observed away from the electrode surface.

Figure 3a presents cyclic voltammograms for Ru­(bpy)_3_^2+^ at concentrations of 0, 16, and 250 μM, where ECL emission appears above 1.0 V vs Ag/AgCl, showing a concentration-dependent increase in current. Figure 3b shows transient electrochemical and optical responses from a representative potential step experiment at 1.2 V vs Ag/AgCl, at which a fast ECL emission signal was observed. At a frame rate of 5 frames per second, the ECL onset appeared immediate, though higher temporal resolution would be required to resolve subsecond kinetics. The ECL signal decays over time as a result of the combination of TPrA depletion (due to the absence of stirring) at the reaction layer and electrode passivation.^19,21,30,57,58^ Oxygen-rich surface moieties are known to be detrimental to the oxidation of TPrA, which in turn will lead to lower emission intensities.^59^

**Figure 3:**
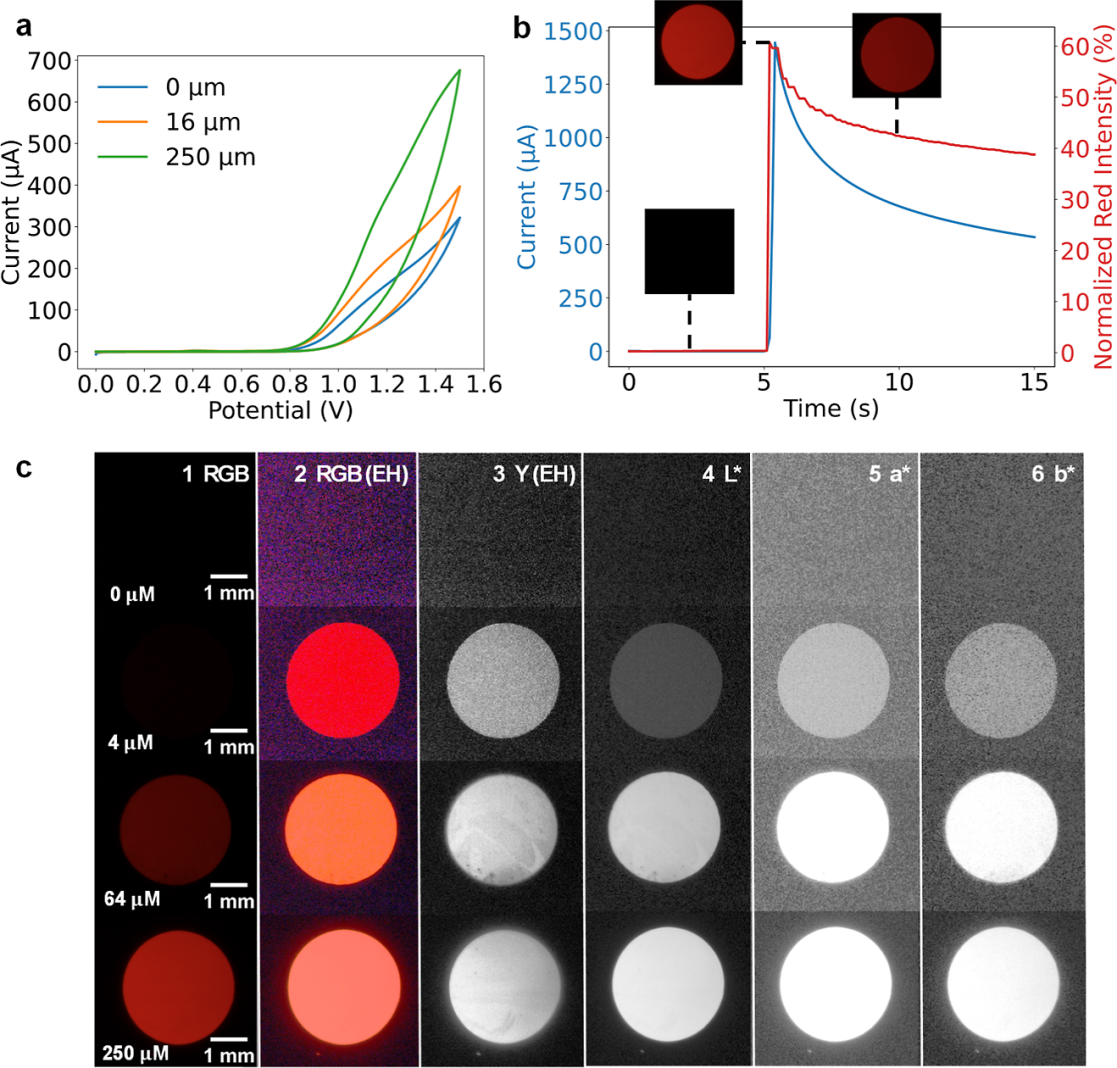
(a) Cyclic voltammetry of different 3 mm glassy carbon disc working electrodes in Ru­(bpy)_3_^2+^ solutions at concentrations of 0, 16, and 250 μM in 180 mM TPrA, 0.3 M phosphate buffer (pH 6.7), with a potential sweep from 0 to 1.5 V vs Ag/AgCl at a scan rate of 50 mV s^–1^. (b) Current response (y_1_, blue) and ECL response as normalized red intensity (%) (y_2_, red) over time during a multistep amperometry experiment, applying 0 V for 5 s followed by 1.2 V vs Ag/AgCl for 10 s, for a representative experiment at 250 μM Ru­(bpy)_3_^2+^. Insets show ECL snapshots at 3, 5, and 10 s (c) ECL signal at Ru­(bpy)_3_^2+^ concentrations of 0, 4, 64, and 250 μM (top to bottom) visualized across different color spaces at the highest intensity point (at ∼5 s): (1) raw RGB gamma-encoded space, (2) RGB with histogram equalization (EH), (3) luminance (Y) from the CIEXYZ color space with EH, and (4–6) L*, a*, and b* channels from the CIELAB color space, respectively. Scale bar: 1 mm.

Similar responses were observed for all concentrations studied, with emission intensities increasing proportionally to concentration, as is appreciable in Figure 3c, which shows a series of images captured at different Ru­(bpy)_3_^2+^ concentrations (0, 4, 64, and 250 μM) during potential step experiments at the highest intensity point (∼5s). Images could reveal spatial inhomogeneities, appearing as darker areas of lower activity (as observed for 64 μM at the bottom left of the electrode surface). Experiments were repeated when the less active areas represented a significant fraction of the electrode area. This stresses the benefits of video-based ECL detection, which provides spatial information that photomultiplier tubes and spectrometers miss. Similar effects have been reported previously.^30,58,60^

### 3.2 Image Analysis for ECL Emission in Different Color Spaces

Figure 3c presents a comparative visualization of the electrode’s ECL emission across various color space channels for representative concentrations (0, 4, 64, and 250 μM), enabling a qualitative analysis of the emission characteristics. Details on the image processing methodology are provided in the Supporting Information Section S1.2. [Figure 3c­(1)] shows the original gamma-encoded RGB images, which look similar for low concentrations. However, ECL information is preserved in the images, as shown in [Figure 3c­(2)] after enhancing contrast through histogram equalization. [Figure 3c­(3)] shows luminance (*Y*) obtained from the CIEXYZ color space after histogram equalization.

Last, [Figure 3c­(4–6)] displays the L*, a*, and b* channels of the CIELAB color space. Notably, the CIELAB representation does not require histogram equalization for visualization, highlighting its high sensitivity to subtle variations in light intensity and its perceptual uniformity, effective even at low luminophore concentrations.

After this qualitative analysis, a quantitative analysis was performed following the methods described in Section 2.5. Figure 4 shows device-dependent RGB color data as a function of Ru­(bpy)_3_^2+^ concentration, whereas Figure 5 examines device-independent CIE transformations.

**Figure 4:**
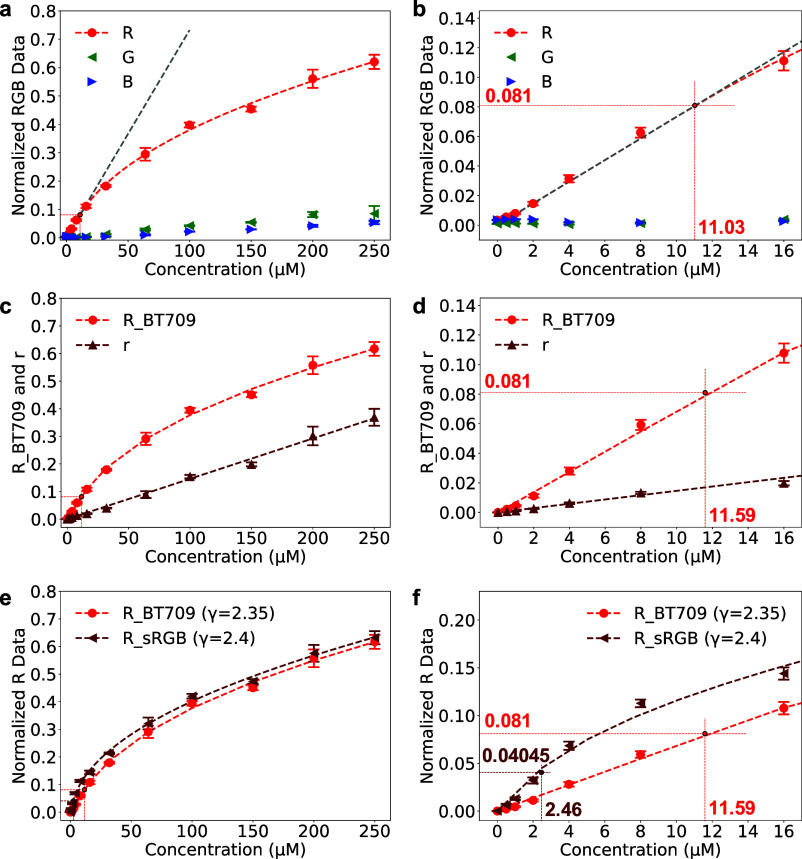
RGB-based ECL signal analysis across Ru­(bpy)_3_^2+^ concentrations (0, 0.5, 1, 2, 4, 8, 16, 32, 64, 100, 150, 200, 250 μM). (a) Normalized RGB data encoded with BT709 gamma correction fitted for R; the gray dotted line indicates an artificial extension of the linear segment. (b) Inset of (a). (c) R-channel data encoded with BT709 gamma correction (R) and after linearization (r). (d) Inset of (c). (e) R-channel data encoded with BT709 and sRGB gamma curves. (f) Inset of (e). All data are plotted against concentration with the 0 μM background signal subtracted in (c–f). Data points represent the mean, error bars indicate the standard deviation for *n* = 3, and dotted lines represent the experimental fit. Threshold markers indicate the transition between linear and power-law regimes, with the corresponding threshold values noted in each inset panel.

**Figure 5:**
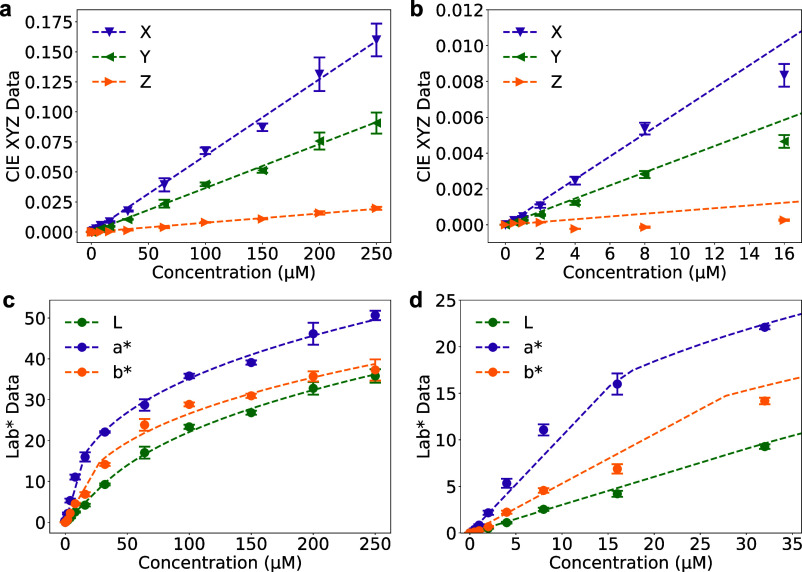
ECL data across several Ru­(bpy)_3_^2+^ concentrations (0, 0.5, 1, 2, 4, 8, 16, 32, 64, 100, 150, 200, 250 μM) represented for CIE device-independent color spaces. (a) Linear CIEXYZ components. (b) Inset of (a). (c) CIELAB channels. d Inset of (c). All data is plotted as a function of concentration, with the background signal (0 μM) subtracted. Data points represent the mean, error bars indicate the standard deviation for *n* = 3, and dotted lines represent the experimental fit.

Figure 4a presents the gamma-encoded intensity values for the RGB channels, extracted from the highest-intensity frame of each BT709-encoded video (typically around 5 s, as illustrated in [Figure 3c­(1)], and plotted against the Ru­(bpy)_3_^2+^ concentrations. As Ru­(bpy)_3_^2+^ emits predominantly in the red spectral region (∼620 nm), the R channel exhibits the strongest response, while the G and B channel signals remain minimal across the studied concentration range. A slight increase in the green channel signal at higher intensities is attributed to spectral overlap with the red filter review [Figure 1b­(1)]. Although all three RGB channels are required for accurate color space transformations, only the red channel was used for ECL signal quantification in this study due to its superior sensitivity.

To model the dependence of the red channel intensity on Ru­(bpy)_3_^2+^ concentration, the gamma function given in eq 3, was adapted to describe the tristimulus nonlinear ECL response

5 $$R=\{\begin{array}{l} b\cdot C,\;C<T\\ {c\cdot C}^{1/\gamma }-d,\;C\geq T \end{array}\;,with\;c=\left(b\cdot C+d\right)/\left(C^{1/\gamma }\right)$$

In this equation, *R* denotes the normalized red channel intensity (R/255), and *C* is the concentration of Ru­(bpy)_3_^2+^. The parameter *T* represents the threshold concentration at which the relationship shifts from a linear to a power-law behavior. The constants *b*, *c*, *d*, and γ are fitting parameters obtained through nonlinear regression using Python (as described in the Supporting Information Section S1.4). The parameter γ defines the exponent of the power-law segment, while *c* is calculated from the other parameters to ensure continuity of the function at *C* = *T*.

For BT709 gamma encoding, given its nominal transfer function threshold of 0.081, the fit was performed using a fixed value of *T* = 11.03 μM. This value corresponds to the approximate concentration, estimated by linear interpolation, at which the red channel transitions from linear to nonlinear response within the experimental range, as represented in Figure 4b. Beyond this point, gamma-induced nonlinearity increases with concentration, requiring correction across the full dynamic range for accurate quantification.

The resulting fit parameters are summarized in Table 1, with an *R* ^2^ value of 0.9986, indicating excellent agreement between the experimental data and the model. The fitted γ value of 2.25 ± 0.24 closely matches the nominal gamma of 2.2 used in the BT709 transfer function, falling within expected experimental and implementation-related variations. It is important to emphasize that applying arbitrary polynomial fits to gamma-encoded data lacks physical meaning and can lead to misleading interpretations.

**Table 1:** Fitting Parameters and Linear Thresholds for the Different Color Space Transformations

data	γ	*b* (μM–1)	*c* (μM^–1^/*n*)	d	*R* ^2^	T (μM)
R_BT709	2.25 ± 0.24	0.00732 ± 0.00072	0.0618 ± 0.0053	0.099 ± 0.042	0.9986	11.02
R_BT709-background	2.29 ± 0.25	0.00685 ± 0.00068	0.0654 ± 0.0056	0.111 ± 0.044	0.9986	11.59
R_sRGB -background	2.14 ± 0.15	0.0181 ± 0.0030	0.0504 ± 0.0071	0.032 ± 0.019	0.9982	2.46
r-background		(14.60 ± 0.20)·10^–4^			0.9964	
*X*-background		(6.359 ± 0.083)·10^–4^			0.9968	
*Y*-background		(3.659 ± 0.049)·10^–4^			0.9971	
*Z*-background		(0.769 ± 0.018)·10^–4^			0.9905	
L*-background		0.301 ± 0.015	8.51 ± 0.20	17.40 ± 0.97	0.9984	27.73
a*-background		1.040 ± 0.048	8.69 ± 0.33	5.2 ± 1.5	0.9962	16.13
b*-background		0.530 ± 0.032	7.35 ± 0.43	7.5 ± 2.1	0.9935	27.73

To linearize the encoded red values, we applied the inverse BT709 transfer function eq 1, using a gamma value independently calibrated via a LEGO bricks-based method^9^ (Figure S1), which yielded γ = 2.35 ± 0.29 (*n* = 5). This value, also consistent with the experimental and nominal values, was preferred for further corrections because it was obtained from a known reference.

Normalized data were background-subtracted using the average signal from *n* = 3 measurements at 0 μM Ru­(bpy)_3_^2+^. Figure 4c compares the gamma-encoded (R) and linearized (r) red channel data. For the encoded R values, background-subtracted data were fitted using eq 5 with a fixed threshold of *T* = 11.59 μM, slightly higher than in previous fits due to the subtraction step. In contrast, the linearized r values were fitted with a simple linear model, *r* = *mC*, enabled by background correction. All fitting parameters are reported in Table 1. In both cases, *R* ^2^ values near 1 confirm the accuracy of the fits and the validity of the linear approximation after gamma correction.

In RGB color spaces within the same luminant (here D65), linearized rgb values remain consistent across encoding variants, whereas gamma encoding functions may differ. Direct conversion between RGB spaces was performed using the linear r values (eq 3). Chromatic adaptation methods can be applied when converting between color spaces with different illuminants to preserve color constancy,^31,32^ but were not required here (as we focused on D65-based color spaces).

Figure 4e compares the BT709 and sRGB transformations, highlighting their differences. The BT709 fit was described previously, while the sRGB fit was obtained using eq 5 again, with the coefficient *T* fixed at 2.46 μM to reflect the lower transition threshold (0.004045) in the inverse sRGB gamma function. The resulting γ value of 2.14 ± 0.15 is close to the nominal 2.4 used in the sRGB transformation (eq 3). The observed differences highlight the importance of using an external, well-characterized reference system instead of relying on experimental data for accurate linearization. The slope of the linear segment for sRGB appears steeper than that of BT709, as expected from the nominal values of the gamma functions.^33^

Despite differences in linear segment slopes and transition thresholds, the gamma-encoded regions of both color spaces are closely aligned, due to the similarity in their respective gamma values.

Figure 5a,b show the transformation of RGB data to the linear device-independent CIEXYZ color space (eq 2), fitted to *XYZ* = *mC*. Fitting parameters are shown in Table 1, presenting an *R* ^2^ close to 1.

Figure 5c,d show the transformation to CIELAB. Here, the functions presented in eq 4 were expressed as a function of Ru­(bpy)_3_^2+^ concentration by an approximated piece-wise function similar to eq 5, used for the RGB encoding

6 $$Lab^{*}=\{\begin{array}{l} b\cdot C,\;C<T\\ {c\cdot C}^{1/3}-d,\;C\geq T \end{array}\;,with\;c=\left(b\cdot C+d\right)/\left(C^{1/3}\right)$$

In this case, a power function with an exponent of 1/3 was used to model the specific CIELAB nonlinear behavior accurately. The fitting parameters are summarized in Table 1, presenting *R* ^2^ values close to 1 stress the excellent agreement between the data and the shape of the fitted functions. Threshold values *T* for L*, a*, and b* were determined using linear interpolation based on the piecewise functions’ thresholds in the CIELAB transformation from *XYZ* (eq 4). For L* and b*, which depended on *Y*/*Y*_*n*_, a threshold of 27.73 μM was calculated using the limiting condition *Y*/*Y*_*n*_ = ${\left(\frac{6}{29}\right)}^{3}$ , the nominal threshold that separates the linear and power law regions in the CIELAB transformation. Similarly, for a*, which depended on *X*/*X*_*n*_, a threshold of 16.13 μM was determined.

### 3.3 Image-Based ECL Quantification Across Color Spaces

ECL quantification through image data is possible beyond the RGB color space. This section explores their benefits and limitations toward a more precise determination of analyte concentrations at the working electrode. Key analytical parameters, including linear dynamic range sensitivity, limit of detection and limit of quantification, were assessed. Details on the statistical data analysis can be found in the Supporting Information Section S1.4.

Three main approaches can be considered for concentration quantification: (i) using the linear region of encoded color spaces, (ii) applying linearization, and (iii), using nonlinear modeling of the encoded values. Of these, the latter 2 cover wider concentration ranges, thereby ensuring broader applicability.

Focusing on linear function-based quantification, Table 2 and Figure 6 summarize the key parameters for the transformations analyzed in this study. These include the gamma-encoded linear segments of R_BT709 and R_sRGB, the linearized r channel, the *X* tristimulus value (CIEXYZ) color space, and the linear segment of a* (CIELAB). These transformations were selected because they best capture the red-emitting Ru-based ECL system. Figure S2 presents the corresponding plots of linear regression on background-subtracted data (*Data* = *mC*).

**Table 2:** Comparison of Color Space Transformations for ECL Linear Quantification^a^

data	linear range (μM)	sensitivity(μM^–1^)	LOD (μM)	LOQ (μM)	recovery (%)	Relative error (%)
R_BT709 · 100	1.44–10.54	0.719 ± 0.023	1.956 ± 0.065	6.52 ± 0.22	96–108 (103)	–4–7 (3)
R_sRGB · 100	2.65–2.46	1.524 ± 0.079	2.65 ± 0.14	8.84 ± 0.47		
r · 100	2.141–250	0.14602 ± 0.00200	2.141 ± 0.034	7.13 ± 0.11	78–118 (97)	–29–15 (−4)
X · 100	3.566–250	0.06359 ± 0.00083	3.566 ± 0.049	11.89 ± 0.16	75–116 (95)	–33–14 (−6)
a*	2.19–16.13	1.087 ± 0.065	2.19 ± 0.13	7.31 ± 0.44	85–133 (110)	–18–25 (6)

a Linear range, sensitivity, limit of detection (LOD), and Limit of Quantification (LOQ) for R_BT709, r, R_sRGB, X, and a* linear segments, indicating estimated values and standard deviations. Recovery (%) and relative error (%) values above LOQ, with observed ranges and corresponding averages provided in parentheses.

**Figure 6:**
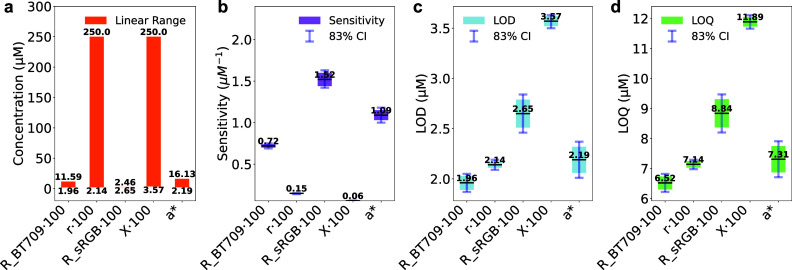
Analytical performance comparison of color spaces for linear response modeling. Dynamic ranges (a) and statistical comparison of sensitivity (b), LOD (c), and LOQ (d) for R_BT709 · 100, r·100, R_sRGB·100, X·100, and a* linear segments. The central dark lines represent the estimated values, while the bar size corresponds to standard deviations (b–d). Light blue error bars indicate 83% confidence intervals (CI), where overlap suggests the possibility of nonstatistically significant differences at a confidence level of α = 0.05. The sample size for blank measurements was *n* = 3.

Linear ranges (Table 2, Figure 6a) are extended from the LOD up to the threshold values of the linear segment, when applicable. The r and X channels show the broadest dynamic range, up to 250 μM, while R_BT709, a* and R_sRGB were limited to the threshold values, 11.59 μM, 16.13 μM and 2.46 μM, respectively. Proving R_sRGB reliable only at very low concentrations, beyond which its response becomes nonlinear.

Sensitivity analysis (Table 2 and Figure 6b) showed a* and R_sRGB as the most sensitive parameters. R_BT709 followed, while *r* and *X* were significantly less sensitive (nonoverlapping 83% CI), highlighting a trade-off between dynamic range and sensitivity.

LOD and LOQ values (Table 2 and Figure 6c,d) ranged from ∼2 to 4 μM and ∼6 to 12 μM, respectively. R_BT709, r, and a* had the lowest detection limits, with overlapping 83% CI indicating no statistical differences, closely followed by R_sRGB (which does not present a valid linear range within this work). *X* exhibited the highest value (worst), which is attributed to its lower sensitivity compared to the other channels.

These estimates are conservative, LOD and LOQ were derived from the average standard deviation of blank samples (*n* = 3), calculated with the python program and propagated through the same transformations as the data, ensuring cumulative error was properly accounted for, toward a robust and realistic estimate of the detection and quantification limits. A detailed description of the LOD and LOQ calculation is provided in the Supporting Information Section S1.4.

To preserve spatial detail, no image smoothing or denoising was applied. Camera settings were manually optimized to avoid signal saturation across a wide concentration range. While this prioritizes reproducibility and robustness, alternative parameters may improve detection limits under specific conditions.

Model accuracy was assessed via relative error and recovery (as defined by equations S4 and S5, Supporting Information). For concentrations above LOQ, relative errors remained within |4–33%| (Figure S3). Recovery values ranged from 75–133% (Figure S4), indicating overall high accuracy. Below the LOD, both relative errors and recovery values deteriorated significantly due to limitations in signal detection. In some cases, relative errors exceeded 100%, reflecting inherent physical constraints affecting histogram intensity, such as pixel size and the Bayer filter.

Although quantification is typically restricted to the linear segment, nonlinear modeling of encoded values can also yield accurate results, provided the color space, transfer function shape, and threshold point are known. Gamma and transformation constants can be captured by the fitting model and may not need to be explicitly defined.

Focusing on nonlinear function-based quantification, Figure 7 and Table 3 summarize the results from previously described nonlinear fits applied to *R* (eq 5) and a* (eq 6). LOD and LOQ were computed using the linear segment within the nonlinear function, and sensitivity refers to the slope of this segment. Because sensitivity is derived from a linear fit by definition, the overall sensitivity of the complete nonlinear function cannot be assessed.

**Figure 7:**
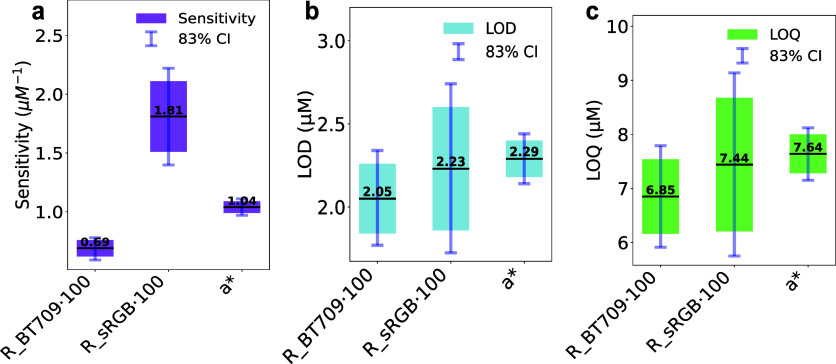
Analytical performance comparison of color spaces for nonlinear response modeling. Statistical comparison of sensitivity (a), LOD (b), and LOQ (c) for R_BT709 · 100, R_sRGB·100 and a* nonlinear modeling. The central dark lines represent the estimated values, while the bar size corresponds to standard deviations. Light blue error bars indicate 83% confidence intervals (CI), where overlap suggests the possibility of nonstatistically significant differences at a confidence level of α = 0.05. The sample size for blank measurements was *n* = 3.

**Table 3:** Comparison of Color Space Transformations for ECL Nonlinear Quantification^a^

data	sensitivity (μM^–1^)	LOD (μM)	LOQ (μM)	recovery (%)	relative error (%)
R_BT709 · 100	0.685 ± 0.068	2.05 ± 0.20	6.85 ± 0.69	89–120 (101)	–8–12 (0)
R_sRGB · 100	1.81 ± 0.30	1.59 ± 0.55	7.4 ± 1.2	83–126 (101)	–11–13 (0)
a*	1.040 ± 0.048	2.29 ± 0.11	7.64 ± 0.36	84–139 (102)	–13–28 (2)

a Sensitivity, limit of detection (LOD), and limit of quantification (LOQ) for R_BT709, R_sRGB and a* nonlinear modeling, indicating estimated values and standard deviations. Recovery (%) and relative error (%) values above LOQ, with observed ranges and corresponding averages provided in parentheses.

Trends remained consistent: R_sRGB showed the highest sensitivity, followed by a* and R_BT709. Overlapping 83% CIs for LOD (∼2 μM) and LOQ (∼7 μM) indicate no significant differences. Above LOQ, relative errors remained below 28%, and recovery values were close to 100% (Figures S3 and S4), presenting an overall improved accuracy.

These values could be considered acceptable, but they might be improved by working on narrower concentration ranges and other image capture conditions.

## 4 Conclusions

This work introduces a general and versatile methodology for quantitative image-based analysis using gamma-encoded color data from digital cameras, including smartphones. By standardizing image acquisition parameters, correcting for nonlinear gamma encoding, and applying appropriate color space transformations, we provide a robust framework for accurate and reproducible quantification across a wide range of applications.

The methodology was demonstrated using electrochemiluminescence (ECL) from Ru­(bpy)_3_^2+^ as a model system. Synchronization of electrode potential, current generation, and image capture enabled precise correlation of emission intensity with experimental conditions, capturing both spatial and temporal aspects of signal evolution.

To ensure reproducibility, all imaging settings (ISO, exposure, focus, white balance, color space) were manually fixed, avoiding variability from automatic adjustments. Automatic camera settings can introduce variability, compromising the reliability of quantitative analyses. Image data were processed using standardized transformations of colorimetric channels (RGB, CIEXYZ, CIELAB), starting with inverting the gamma-encoding for data linearization. This allowed for device-independent, statistically validated quantification of image intensity data.

The proposed framework supports both linear and nonlinear quantification models, whose performance was assessed through sensitivity, LOD, LOQ, recovery, and relative error. Linearized channels such as r (RGB) and X (CIEXYZ) demonstrated wide dynamic ranges and acceptable sensitivity, while working with gamma-encoded R (BT709, sRGB) and a* (CIELAB) linear segment regions offered enhanced sensitivity for low-concentration signal detection, necessitating nonlinear models to increment the detection range.

Importantly, quantification performance depends on the choice of encoding standard (BT709 vs sRGB), as their different gamma functions affect linearization and signal accuracy, particularly in low-light regions. Choosing the appropriate encoding ensures reliable interpretation of luminophore emissions in video (BT709) or image (sRGB) data, highlighting the need for tailored transfer functions and modeling strategies.

Above the LOQ, relative errors and recovery values were, on average, lower than 6% and around 100%, respectively, presenting, in general, a higher accuracy when using nonlinear models. While smoothing algorithms could further reduce noise, they were excluded to preserve spatial fidelity.

Although the methodology was demonstrated with a red-emitting luminophore, it is readily transferable to other emission colors and optical systems. Channels R and r (red from RGB), X (CIEXYZ), and a* (CIELAB) offered optimized detection across various concentration ranges for the red-emitting Ru-based system examined here. In contrast, for luminol-based systems emitting in the blue region (∼425 nm),^61^ gamma-encoded and linearized blue from RGB, *Z* (CIEXYZ), and b* (CIELAB) will likely become more relevant.

More importantly, this approach is applicable beyond ECL, any system involving gamma-encoded image data can benefit from this framework, such as colorimetric sensors, fluorescence imaging, or raw-to-compressed image conversion. It is particularly well-suited to low-cost, portable platforms using commercial camera sensors, offering a path toward reliable, high-throughput optical quantification in analytical chemistry, biosensing, and diagnostics, where data interoperability is most important.

## Supplementary Material



## Data Availability

The experimental data and Python scripts supporting this study are openly available via AMS Acta at 10.6092/unibo/amsacta/8391. In addition, the scripts can be accessed through the ECLectic GitHub repository, branch: “Gamma Correction and Color Space Transformations”.

## Notes

It includes, in PDF format, extended experimental methods detailing video acquisition and export parameters, manual visualization of video data, determination of the digital gamma parameter using a reference color set, and evaluation of the ECL detection system, including statistical analyses. Additional figures are provided to support the main findings, including linear calibration fits of ECL signal versus Ru­(bpy)_3_^2+^ concentration across different color spaces, relative error analysis, and recovery values throughout the studied concentration range (PDF)
