# Supplementary material for: Gamma Correction and Color Space Transformations for Quantitative Analysis of Electrochemiluminescence Images Using Smartphone Cameras
Source: Chem Biomed Imaging. 2025 Jun 26;3(11):767–78. doi: 10.1021/cbmi.5c00056 (PMC12648430; doi:10.1021/cbmi.5c00056)
Supplement: Supplementary file 1 [file im5c00056_si_001.pdf]

Supporting Information

Gamma Correction and Color Space Transformations for Quantitative Analysis of Electrochemiluminescence Images Using Smartphone Cameras

Stephania Rodríguez Muiña [a], *Rajendra Kumar Reddy Gajjala* [a],

Eduardo Fernández Martín [a] *and Francisco Javier del Campo* [a,b*]

[a]BCMaterials, Basque Center for Materials, Applications and Nanostructures. UPV/EHU Parque Científico, E-48940 Leioa, Bizkaia, Spain.

[b] IKERBASQUE, Basque Foundation for Science, 48009 Bilbao, Spain.

Email: Javier.delcampo@bcmaterials.net

Table of contents

1. Methods Supplementary Information 3
   1.1 Video acquisition and exporting parameters 3
   1.2 Video data manual visualization 6
   1.3 Determination of the digital gamma parameter using a reference color set 8
   1.4 Evaluation of the ECL detection system and statistical data analysis 10
2. Results Supplementary Plots 14

1. Methods Supplementary Information

1.1 Video acquisition and exporting parameters

Video recordings were captured using the *MotionCam Pro* smartphone application (version 3.5.5-pro, Play Store). The following acquisition parameters were selected to ensure consistent image quality across experiments:

- A resolution of 1920×1080 pixels. The resolution defines the video's frame size, 1920×1080 balances image clarity and detail against storage and processing demands, and offers a widely compatible, high-quality standard. It could be increased for greater detail if storage and hardware allow it.
- Fixed focus for consistent image sharpness.
- A frame rate of 1/5 s (5 frames per second -fps-). The frame rate determines how frequently the camera captures a new image over time. Lower frame rates like 5 fps allow each frame to collect more light, enhancing brightness in low-light settings like electrochemiluminescence (ECL), though at the cost of reduced temporal resolution.
- Equivalent ISO 100. ISO defines the sensor's sensitivity to light. Lower ISO values (e.g., 100) reduce noise and preserve detail. ISO 100 was chosen to maintain image quality across a wide concentration range; higher ISO settings (e.g., 800) may be used for lower concentrations if the signal-to-noise ratio permits.

After acquisition, videos were rendered directly within the app using the export settings detailed in Table S1. These parameter choices are designed to preserve the integrity of ECL data while ensuring reliable video comparisons in this study. The table explains the benefits, drawbacks, and alternative settings, allowing researchers to select the most suitable parameters based on their specific experimental requirements.

Table S1. ECL video export parameters for scientific data comparison [1–5].

Parameter	Definition	Chosen Value	Benefits	Drawbacks	Alternatives
**Look-Up Table (LUT)**	LUTs modify color grading.	No LUT	Ensures accurate intensity representation.	No visual enhancement.	Use a scientifically calibrated LUT if correction is needed.
**Gain**	Gain amplifies signal intensity (can introduce noise).	+0 EV	Maintains original signal intensity.	No signal amplification, which may limit detection of low-intensity emissions.	Increment for improving signal in ultra-low light conditions, always adapted to avoid overexposure in high-intensity regions.
**Shadows**	Adjust dark areas.	0 %	Maintains original signal intensity.	Could obscure low-intensity signals.	Increase slightly to improve contrast in dim areas without introducing artifacts.
**Contrast**	Adjusts the difference between light and dark regions.	0 %	Keeps original intensity relationships.	May reduce differentiation in data.	Adjust only if necessary for signal visibility.

**White Point / Black Point**	Defines intensity range.	0 / 0 %	Prevents artificial contrast changes.	May limit enhancement for better visibility.	Adjust if histogram compression or data clipping is observed.
**Saturation**	Controls the intensity of colors.	1.00	Preserves the native color intensity without artificial enhancement.	May limit visualization enhancements, especially if subtle color variations are informative.	Increase slightly if improved contrast or color differentiation is needed for analysis.
**Sharpness**	Enhances edge definition.	0 %	Avoids artificial edge distortion.	May cause blurring of small structures.	Increase if necessary for clarity.
**Detail**	Enhances small feature visibility.	0 %	Preserves original structure.	May make faint signals harder to see.	Adjust slightly if necessary for feature visibility.
**Temperature**	Adjusts color balance.	6500 K	Provides standard neutral white balance.	Might not match specific camera settings.	Adjust if environmental correction is needed.
**Tint**	Modifies color cast.	0 %	Ensures no artificial color tinting.	May not match original camera setting.	Adjust slightly if needed for accuracy.
**Noise Reduction (Temporal, Motion Threshold, Spatial, Chroma)**	Reduces unwanted variations in signal intensity.	0	Prevents signal distortion.	Might leave visible noise in the video.	Increase noise reduction by combining frames, increasing intensity threshold or denoising algorithms if necessary.
**Video Codec**	Compression algorithm for video storage and playback.	H.265	Efficient compression with high image quality and	Higher computational requirements	ProRes or FFV1 for lossless quality; H.264

			smaller file sizes.	for encoding and playback.	for broader compatibility.
**Transfer Function**	Describes how scene-referred linear light values are encoded into non-linear values for storage and display (e.g., gamma curves or color space transformations).	BT709	Standard color profile for accurate brightness mapping.	May clip data if original recording is in a linear profile.	Select the appropriate color space encoding for your application; choose a linear color space when possible to preserve true intensity relationships for scientific analysis.
**Maximum Bit Rate**	Determines the amount of data stored per second.	60 Mbps	Maintains sufficient quality for intensity preservation.	File sizes may be large.	Increase to 80–100 Mbps if pixelation occurs.
**Key Frame Interval**	Sets how often a full (intra) frame is stored in the video stream.	60	Balances compression efficiency and seekability; frequent key frames help maintain video consistency.	Larger file sizes compared to longer intervals.	Use lower values for scenes with rapid changes to improve accuracy; longer intervals for static content to reduce size.
**Bit Depth**	Defines the number of intensity levels per channel.	8-bit	Standard format for video storage.	May not capture subtle intensity changes.	Use 10-bit or 12-bit if available.
**Pixel Format**	Defines how much color information is stored per pixel.	4:2:0	Reduces file size with moderate quality retention.	Some color data is lost.	Use 4:2:2 or 4:4:4 for better color accuracy.

1.2 Video data manual visualization

Manual data analysis was carried out using VLC media player and ImageJ for careful and precise inspection. Individual frames corresponding to the highest ECL intensity, occurring at the potential step, were extracted using the *Take Snapshot* tool in VLC Media Player. The frames were saved in .png format to preserve the original color space. VLC Media Player can be used as well to verify detailed codec information for video files, including resolution, encoding format (such as H.265), frame rate, and color space.

Extracted frames were analyzed in FIJI ImageJ [6]. For each frame, the electrode region of interest was selected manually, and the Color Histogram Tool was used to obtain the mean and standard deviation of the RGB intensity values within this region. These quantitative measurements served to verify the results produced by the custom Python program (available at GitHub branch for Gamma Correction and Color Space Transformations). Additionally, color space transformation (RGB to Luminance and RGB to CIELAB), and Enhance Contrast (selecting Equalize Histogram option, which ignores saturated pixel percentage) were applied for qualitative inspection and visualization purposes.

Histogram equalization (HE) was applied for data visualization and to identify the electrode area for further analysis at concentrations where the red emission was not visually apparent (0 – 8 μM). HE is an image processing technique that enhances contrast by redistributing pixel intensity values across the full intensity range. This process expands the most frequent intensity levels, making low-contrast regions more distinct and improving overall visibility and detail.

Nevertheless, all quantitative analyses were performed on the original, unprocessed data using an automated custom Python program (available at GitHub branch for Gamma Correction and Color Space Transformations) to ensure accuracy.

1.3 Determination of the digital gamma parameter using a reference color set

To determine the gamma value applied under the specific experimental conditions, a reference set of four LEGO® bricks (white, medium stone grey, dark stone grey, and black) with known sRGB values [7] was used (see Table S2). These bricks were recorded using a standard light source under the selected experimental parameters (except for a frame rate of 30 fps instead of 5 fps to avoid saturation), yielding BT709-encoded RGB values. RGB experimental intensities were measured by selecting a representative frame using VLC Media Player and analyzing each LEGO® color region with the Color Histogram tool in ImageJ, following the same manual methodology described above. Since a perfect linear correlation was expected between the recorded results and the sRGB values, the gamma value was iteratively adjusted using Excel's Solver (Generalized Reduced Gradient Nonlinear method) so that the correlation coefficient, R^2, was as close as possible to 1 [8].

Luminance (Y from CIEXYZ) was then calculated for both datasets, following the mathematical procedure explained in the Methods section 2.5:

- For the sRGB reference values, the standard gamma correction of 2.4 was applied.
- For the BT709-encoded experimental values, an initial arbitrary gamma value was used for correction, as its exact value under these conditions was unknown.

To determine the correct gamma value for BT709, the luminance values from both datasets were plotted against each other in Excel. The R^2 value of the resulting linear fit was calculated using the LINEST function. Since the ideal relationship should exhibit a linear correlation (as Y is a linear parameter), the gamma value for BT709 was iteratively adjusted using the Solver function (GRG Nonlinear method) to maximize R^2 and achieve the best possible linearity. This approach provided a robust calibration method, minimizing errors associated with direct experimental data fitting.

The obtained fit is shown in Figure S1, the slope differs from 1 because the illumination used in the determination of gamma differed from illuminant D65, used as standard in sRGB. In the case of the present work, where the analyte is the illuminant, we are only concerned with the value of gamma applied in the conversion. The found gamma value was 2.35 $\pm$ 0.29 (n=5).

Table S2. Colorimeter LEGO pieces data for sRGB and CIELAB [7].

LEGO piece	sRGB			CIELAB		
	R	G	B	L*	a*	b*
**White**	244	238	228	94	1	6
**Medium Stone Grey**	160	163	164	67	-1	-1
**Dark Stone Grey**	98	101	102	42	-1	-1
**Black**	18	18	21	5	1	-2

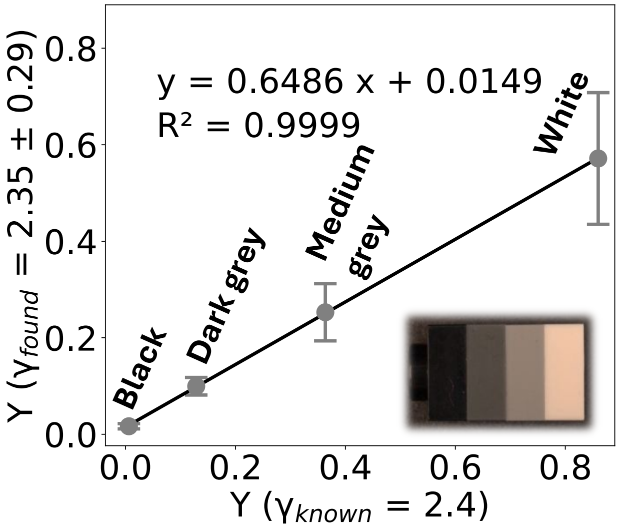

Figure S1. Determination of gamma using LEGO® brick luminance values. The x-axis represents the calculated luminance based on literature's sRGB values for the LEGO® bricks (Table S2), while the y-axis shows the luminance of the experimental data, for which the gamma value was iteratively adjusted to maximize R^2. Fit data is displayed (slope of 0.6486 ± 0.0038, intercept of 0.0149 ± 0.0028 and R^2 of 0.9999). Gray data points indicate mean values, with error bars representing the standard deviation (n = 3). The inset displays an experimental snapshot of the four LEGO® bricks used (black, dark grey, medium grey, and white).

1.4 Evaluation of the ECL detection system and statistical data analysis

Following the described color transformations of the methods section 2.5, the extracted color space channel intensities were processed by subtracting the background signal at 0 concentration. Both linearized and non-linearized data were plotted against analyte concentration. Fittings were performed in Python (code available at GitHub branch for Gamma Correction and Color Space Transformations) using the curve_fit function from the scipy.optimize module, which employs

non-linear least squares optimization to minimize the difference between observed and modelled data. This method yields the optimal parameters (popt) to best-fit the model (γ, b, c, d), along with their associated uncertainties derived from the diagonal elements of the covariance matrix (pcov).

To evaluate model performance, the coefficient of determination (R^2) was calculated using the r2_score function from sklearn.metrics, providing a measure of how well the model explains data variability.

The linearized data, along with the linear segments of the nonlinear color spaces and the nonlinear defined fitting models, were evaluated to determine the applicable linear range, sensitivity (defined as the slope m in $y = mx$), and analytical figures of merit such as the limit of detection (LOD) and limit of quantification (LOQ). Following the approach described in [9], using the equation (S1).

$$C_L = \frac{k \times \sigma_{blank}}{m}, \ \Delta C_L = C_L \sqrt{\left(\frac{\Delta\sigma_{blank}}{\sigma_{blank}}\right)^2 + \left(\frac{\Delta m}{m}\right)^2} \qquad (S1, \quad S2)$$

Where the concentration limit (C_L) was calculated using the standard constant k, 3 for LOD and 10 for LOQ [9], the average of the experimental standard deviation of the blank signal σ_{blank} and the slope of the linear segment, m. The term ΔC_L accounts for the propagated uncertainty, calculated as shown in equation (S2), with Δm, being the error of the slope obtained from the fit, and $\Delta\sigma_{blank}$, the blank standard deviation of the experimental error average.

The σ_{blank} values were determined by averaging the standard deviations of color intensity values measured within the mask for blank samples (n=3), obtained using Python. This standard deviation

was then propagated through the same mathematical transformations applied to the actual data, ensuring that the error contributions from these calculations were fully accounted for.

All analytical figures were compared across different color space transformations to identify the most suitable representation for the experimental conditions.

To evaluate the statistical significance of differences between parameters, confidence intervals (CIs) were calculated. Since only a single estimate was available for each case—rather than an average derived from multiple experimental replicas—a conservative approach was taken by setting n = 1 in the standard CI equation. In this adaptation, the sample mean ($\bar{x}$) and t-distribution were replaced with the estimated parameter ($\mathrm{estimate}$) and the standard normal (z) distribution. This modified approach, depicted in Equation (S3), constructs the CI to account for uncertainty despite the absence of multiple data points, ensuring a more cautious interpretation of statistical significance.

$$CI = \bar{x} \pm t_{\frac{\alpha}{2},n-1} \cdot \frac{\sigma}{\sqrt{n}} \quad \xrightarrow{n\,=\,1} \quad CI = estimate \pm (z - value) \cdot \sigma \qquad \text{(S3)}$$

where σ is the standard deviation, and the $z - value$ is derived from the standard normal distribution.

Caution should be taken when interpreting results presented with confidence or standard error intervals. The overlap of these intervals does not necessarily imply statistical significance for the parameters of interest. When using this estimate approach, an 83% confidence interval is the most appropriate for hypothesis testing, as it provides an approximate $\alpha = 0.05$ significance test when

intervals overlap [10,11]. The critical z-value (equation (S3)) for an 83% confidence interval is approximately 1.37.

To assess the model accuracy, the relative error and recovery percentage were calculated. The relative error, expressed as a percentage, quantifies the deviation of the predicted values from the observed values and was computed using the equation (S4):

$$Relative\ error\ (\%) = \frac{Observed\ value - Predicted\ value}{Observed\ value} \times 100 \qquad \text{(S4)}$$

where the $Observed\ value$ corresponds to the experimentally measured result or the value obtained using the specified color space transformations, while the $Predicted\ value$ is determined from the calibration curve as $m{\cdot}C$, with m being the slope of the calibration curve and C the analyte concentration.

Similarly, the recovery percentage compares the predicted concentration to the actual prepared concentration. Although there is no set methodology for the determination of recovery, we followed IUPAC's definition[12] which states that "*Recovery is the proportion of the amount of analyte, present in or added to the analytical portion of the test material, which is extracted and presented for measurement.*", and applied the equation (S5):

$$Recovery\ (\%) = \frac{Predicted\ concentration}{Real\ concentration} \times 100 \qquad \text{(S5)}$$

where the $Real\ concentration$ refers to the known concentration present in the sample, and the $Predicted\ concentration$ is the concentration estimated from the fit as $data/m$, with $data$ being the ECL signal in a given color space at the measured signal level, and m the slope of the calibration curve function.

While recovery values approaching 100% are ideal, conventional thresholds consider values between 80–120% acceptable, 90–110% very good, and 95–105% excellent.

This approach allows for a systematic evaluation of the model's predictive performance across the entire concentration range studied, providing valuable insights into the method's reliability, sensitivity, and potential limitations.

2. Results Supplementary Plots

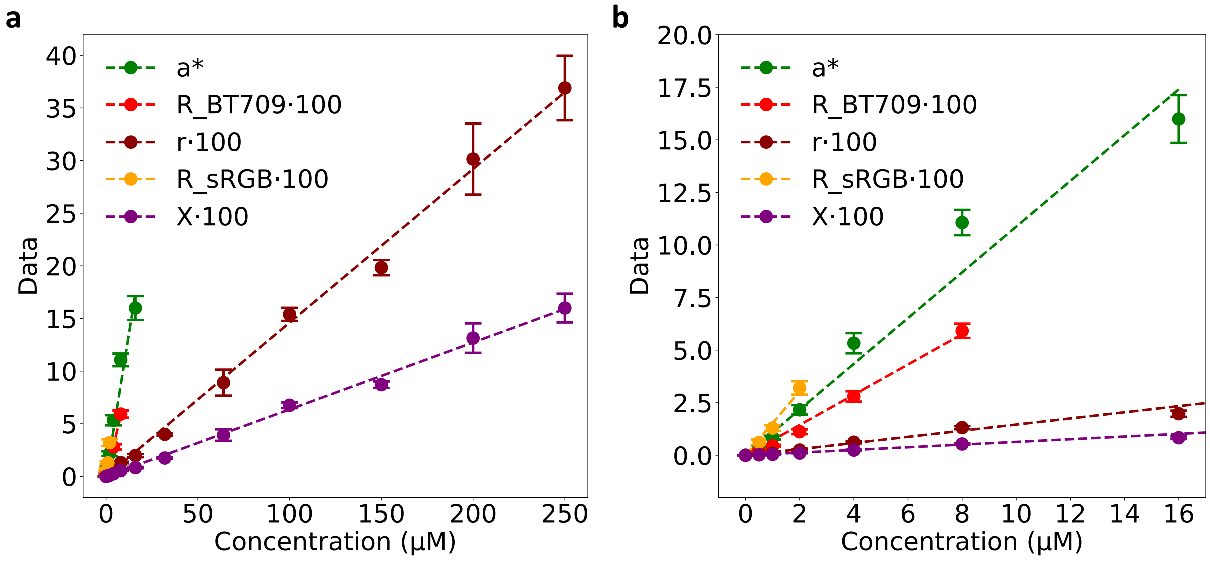

Figure S2. Linear fit of ECL data across several $Ru(bpy)_3^{2+}$ concentrations (0, 0.5, 1, 2, 4, 8, 16, 32, 64, 100, 150, 200, 250 μM) for different color spaces. (a) Linear fit of a*, R_BT709·100, r·100, R_sRGB·100 and X·100 as a function of the concentration. (b) Enlarged view of plot (a), focusing on the low-concentration range. Data points represent the mean, error bars indicate the standard deviation for n = 3, and dotted lines represent the experimental fit.

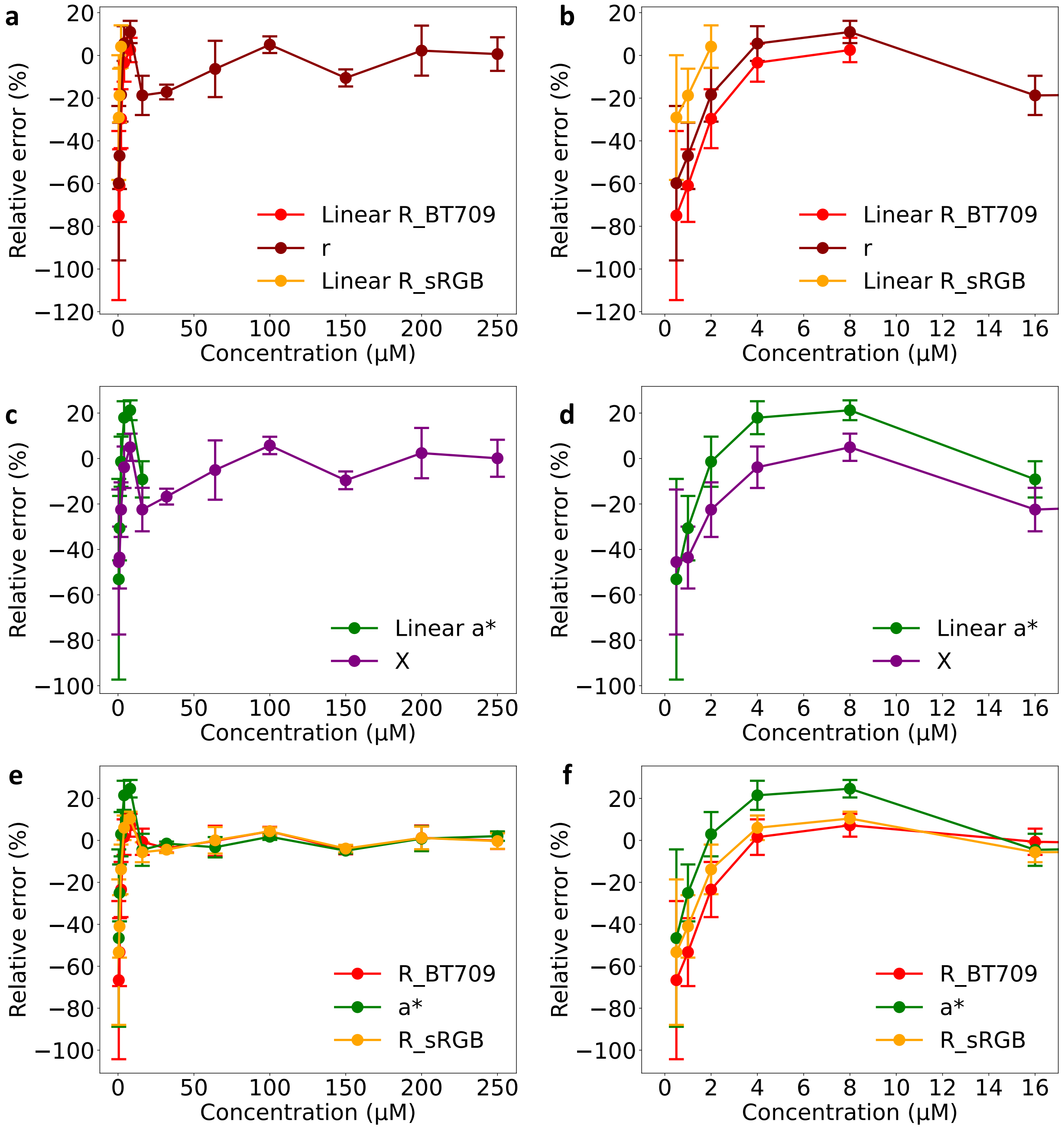

Figure S3. Relative errors represented over the $Ru(bpy)_3^{2+}$ concentration range studied. (a) Relative error (%) for device-dependent color spaces R_BT709·100, r·100, R_sRGB·100 linear segments. (b) Enlarged view of plot (a), focusing on the low-concentration range. (c) Relative error (%) for device-independent color spaces a* and X·100 linear segments. (d) Enlarged view of plot (c),

focusing on the low-concentration range. (e) Relative error (%) for R_BT709, R_sRGB and a* nonlinear modeling. (f) Enlarged view of plot (e), focusing on the low-concentration range. Data points represent the mean, error bars indicate the standard deviation for n = 3, and lines are used solely to connect the points.

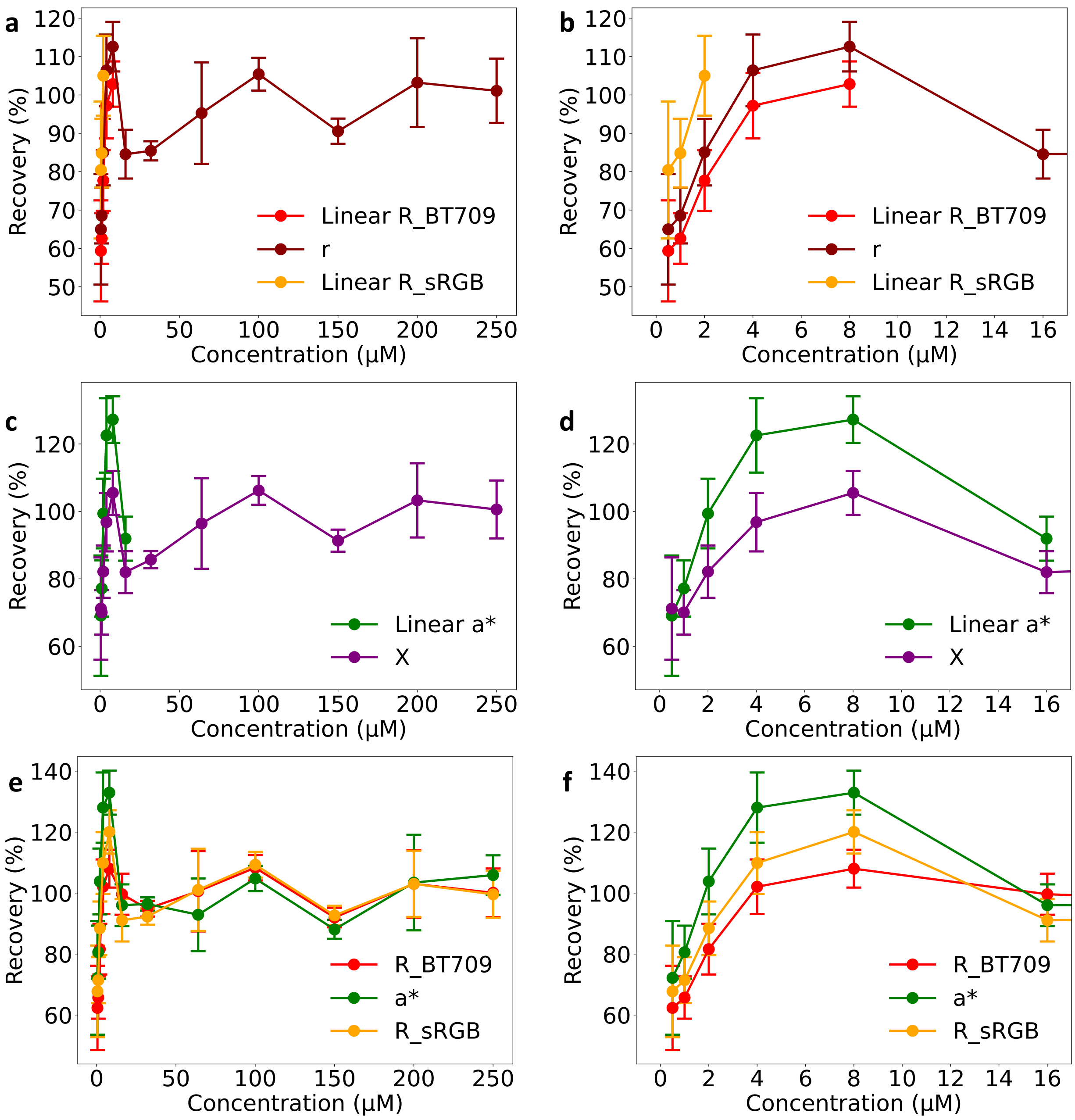

Figure S4. Recovery values represented over the $Ru(bpy)_3^{2+}$ concentration range studied. (a) Recovery (%) for device-dependent color spaces R_BT709·100, r·100 and R_sRGB·100 linear segments. (b) Enlarged view of plot (a), focusing on the low-concentration range. (c) Recovery (%) for device-independent color spaces a* and X·100 linear segments. (d) Enlarged view of plot (c), focusing on the low-concentration range. (e) Recovery (%) for R_BT709, R_sRGB and a*

nonlinear modeling. (f) Enlarged view of plot (e), focusing on the low-concentration range. Data points represent the mean, error bars indicate the standard deviation for n = 3, and lines are used solely to connect the points.

REFERENCES

(1) Poynton, C. A. *Digital Video and HD: Algorithms and Interfaces*, 2nd ed.; Morgan Kaufmann: Waltham, MA, 2012.

(2) Foster, D. H. Color Constancy. *Vision Res.* **2011**, *51* (7), 674–700. https://doi.org/10.1016/j.visres.2010.09.006.

(3) Pouli, T.; Reinhard, E.; Larabi, M.-C.; Abebe, M. A. Chapter 9 - Color Management in HDR Imaging. In *High Dynamic Range Video*; Dufaux, F., Le Callet, P., Mantiuk, R. K., Mrak, M., Eds.; Academic Press, 2016; pp 237–272. https://doi.org/10.1016/B978-0-08-100412-8.00009-7.

(4) Ramanath, R.; Drew, M. S. Color Spaces. In *Computer Vision: A Reference Guide*; Ikeuchi, K., Ed.; Springer US: Boston, MA, 2014; pp 123–132. https://doi.org/10.1007/978-0-387-31439-6_452.

(5) Ebner, M. *Color Constancy*; Wiley-IS&T series in imaging science and technology; J. Wiley: Chichester, 2007.

(6) Schindelin, J.; Arganda-Carreras, I.; Frise, E.; Kaynig, V.; Longair, M.; Pietzsch, T.; Preibisch, S.; Rueden, C.; Saalfeld, S.; Schmid, B.; Tinevez, J.-Y.; White, D. J.; Hartenstein, V.; Eliceiri, K.; Tomancak, P.; Cardona, A. Fiji: An Open-Source Platform for Biological-Image Analysis. *Nat. Methods* **2012**, *9* (7), 676–682. https://doi.org/10.1038/nmeth.2019.

(7) Bartneck, C. *The Unofficial LEGO Color Guide*, 5th ed.; Independently Published, 2022.

(8) Velasco, J.; Galán, P.; Gajjala, R. K. R.; Fernández, E.; Trimaille, J.; Hihn, J.-Y.; Lakard, B.; Álvarez-Gila, A.; Picón, A.; del Campo, F. J. Image-Based Analysis of Electrochromic

Materials: Gamma Correction with a LEGO Luminance Checker. *Electrochimica Acta* **2025**, *525*, 146062. https://doi.org/10.1016/j.electacta.2025.146062.

(9) Long, G. L.; Winefordner, J. D. Limit of Detection. A Closer Look at the IUPAC Definition. *Anal. Chem.* **1983**, *55* (7), 712A-724A. https://doi.org/10.1021/ac00258a001.

(10) Payton, M. E.; Greenstone, M. H.; Schenker, N. Overlapping Confidence Intervals or Standard Error Intervals: What Do They Mean in Terms of Statistical Significance? *J. Insect Sci.* **2003**, *3*, 34.

(11) Austin, P. C.; Hux, J. E. A Brief Note on Overlapping Confidence Intervals. *J. Vasc. Surg.* **2002**, *36* (1), 194–195. https://doi.org/10.1067/mva.2002.125015.

(12) Thompson, M.; Ellison, S. L. R.; Fajgelj, A.; Willetts, P.; Wood, R. Harmonized Guidelines for the Use of Recovery Information in Analytical Measurement. *Pure Appl. Chem.* **1999**, *71* (2), 337–348. https://doi.org/10.1351/pac199971020337.
